# Influence of Off-resonance in myocardial T1-mapping using SSFP based MOLLI method

**DOI:** 10.1186/1532-429X-15-63

**Published:** 2013-07-22

**Authors:** Peter Kellman, Daniel A Herzka, Andrew E Arai, Michael Schacht Hansen

**Affiliations:** 1 National Heart, Lung, and Blood Institute, National Institutes of Health, DHHS, 10 Center Drive MSC-1061, Bethesda, MD 20892, USA; 2 Biomedical Engineering, Johns Hopkins University School of Medicine, Baltimore, MD, USA

**Keywords:** T1 map, Error, Off-resonance, MOLLI, Shim, SSFP

## Abstract

**Background:**

Myocardial T1-mapping methods such as MOLLI use SSFP readout and are prone to frequency-dependent error in T1-measurement. A significant error in T1 may result at relatively small off-resonance frequencies that are well within the region without banding artifacts.

**Methods:**

The sensitivity of T1-estimates based on the SSFP based MOLLI sequence to errors in center frequency are calculated by means of a Bloch simulation and validated by phantom measurements. Typical off-resonance errors following local cardiac shimming are determined by field mapping at both 1.5 and 3.0T. In vivo examples demonstrate the artifactual appearance of T1-maps in the presence of off-resonance variation.

**Results:**

Off-resonance varied 61.8 ± 15.5 Hz (mean ± SD, n = 18) across the heart at 1.5T and 125.0 ± 40.6 Hz (mean ± SD, n = 18) at 3.0T. For T1 = 1000 ms, the variation in T1 due to off-resonance variation was approximately 20 ms at 62 Hz, and > 50 ms at 125 Hz.

**Conclusions:**

Regional variations due to the inability to completely shim the B0-field variation around the heart appear as regional variation in T1, which is artifactual.

## Background

The frequency response of steady state free precession (SSFP) sequences is well known and results in dark band artifacts [1-3]. Myocardial T1-mapping methods such as the modified Look-Locker inversion recovery method (MOLLI) [4,5] use SSFP readout and are therefore prone to frequency dependent errors in T1-measurements. The off-resonance behavior of SSFP and associated banding artifacts are often analyzed assuming the magnetization is at steady state as in continuous cine imaging. However, the MOLLI imaging sequence uses single shot imaging with data acquired on the approach to steady state. As a result, the off-resonance response becomes dependent on the initial condition, which is in turn dependent on the inversion recovery time. This leads to a frequency dependent change in the apparent inversion recovery. It is not well appreciated that a significant error in T1 may result at relatively small off-resonance frequencies that are well within the region without banding artifacts. As T1-mapping and extra-cellular volume (ECV) mapping based on T1 measurement are used to detect more subtle cardiomyopathies [6-14], small errors in T1 become more significant. Hence, even small variations in T1 introduced by off-resonance due to the inability to completely shim B0-field variation may lead to significant biases in measured T1 that may be falsely confused with real pathology.

The readout excitation flip angle used in MOLLI is typically low (35°) compared to SSFP imaging as used for cine function imaging to reduce the influence of the readout on the inversion recovery. We hypothesize that reducing the flip angle improves T1-measurement accuracy with significant effect on the sensitivity to off-resonance. In this work, the sensitivity of T1 measurement to off-resonance frequency and readout excitation flip angle are quantified for specific MOLLI protocols.

## Methods

### Simulation

A waveform level Bloch simulation of the MOLLI T1-mapping method was implemented to study errors in T1-mapping, their dependencies and their sensitivities to various sequence and protocol design parameters. The simulation included the RF excitation pulse waveform and gradients in order to accurately model the variation in flip angle across the slice profile. Simulation results are provided for a specific MOLLI protocol which used a 5(3s)3 sampling scheme (similar to protocol used in [10,11]) which uses 2 inversions with images acquired for 5 heartbeats following the first inversion, followed by a 3 second recovery period, and images acquired for 3 heartbeats following the second inversion. T1 fits are performed to the simulated magnetization using a 3-parameter model and a Look-Locker correction is applied to the apparent T1 to correct for the influence of readout [4,5,15]. The T1-error due to the approximation used in the Look-Locker correction is the predominant error in this analysis [16]. Influence of magnetization transfer (MT) or imperfect inversion [17] are not considered here. Imaging parameters were: 256 × 144 matrix, FA = 35°, TR = 2.8 ms, TImin = 105 ms, TIshift = 80 ms. A partial Fourier acquisition in the phase encode dimension was used to reduce the minimum achievable inversion time and reduced the overall shot duration. A partial Fourier factor of 7/8 was used in the phase encode direction with 126 actual acquired lines (144 matrix size) plus 12 additional central lines for parallel imaging auto-calibration. The center of k-space was at 33 lines and there were 5 additional linear ramp flip angle pulses to reduce transient oscillations [18]. The T1 error was calculated for a range of T1’s from 200-1200 ms at a fixed T2 = 45 ms. The errors were also simulated for the specific values of phantom T1 and T2 used for comparison with measurements.

Additional simulations were performed to assess the sensitivity of off-resonance errors to imaging protocol parameters. Sensitivity to off-resonance arises due to the influence of the SSFP readout during the approach to steady state. Both the total number of RF pulses (Ntotal) and the number of pulses to the center of k-space (Ncenter) will affect the T1-mapping accuracy and sensitivity to off-resonance. The measured phantom and in-vivo protocols used Ncenter/Ntotal: 33/75, based on a matrix size with 144 phase encodes, Fourier factor = 7/8, and 12 extra central lines acquired for in-place auto-calibration of parallel imaging (factor 2). A range of protocols were tested and results are provided for a significantly shortened Ncenter/Ntotal: 16/48, based on a matrix size with 128 phase encodes, Fourier factor = 3/4, and no extra central lines acquired corresponding to a separate reference line approach for auto-calibration.

### Phantom measurements

Experimental validation was performed for a CuSO4 doped agar gel phantom using the specific MOLLI protocol at varying off-resonance frequencies in 10 Hz increments. Measured and simulated T1 are compared for a test tube phantom with T1 = 1197 ms and T2 = 47 ms (1.5T Magnetom AERA, Siemens Medical Solutions, Erlangen, Germany).

### Field map measurements

In-vivo measurements of off-resonance maps due to variation in B0-field were acquired to determine the typical expected variation in frequency. Measurements were made in n = 18 subjects referred for CMR assessment of known or suspected heart disease at both 1.5 (age 46 ± 20) and 3.0T (age 36 ± 17) using a multi-echo GRE sequence. Field maps were estimated as a byproduct of water fat separated image reconstruction [19]. A second order shim was performed over a local volume (box) encompassing the whole heart. Off-resonance frequency variation was measured in the left ventricle for a mid-ventricular, single short axis slice per subject.

### In-vivo imaging

In-vivo T1-maps were acquired for normal healthy volunteers to illustrate the apparent variation in measured T1 with off-resonance. Images were acquired at several center frequencies to demonstrate the sensitivity. Imaging was performed on 1.5T Magnetom AVANTO and AERA scanners and 3T Magnetom SKYRA scanner (Siemens Medical Solutions, Erlangen, Germany). This study was approved by the local Institutional Review Board of the National Heart, Lung, and Blood Institute and all subjects gave written informed consent to participate.

## Results

### Off-resonance variation

Off-resonance frequency in the LV was measured at 1.5 and 3.0T after shimming over a local heart volume. At 1.5T, the mean off-resonance frequency in the LV myocardium was 20.3 ± 13.0 Hz. The maximum off-resonance in the LV was 61.8 ± 15.5 Hz (n = 18). At 3.0T, the mean off-resonance frequency in the LV myocardium was 15.4 ± 29.3Hz. The maximum off-resonance in the LV was 125.0 ± 40.6 Hz (n = 18).

### Simulation

**Figure 1:**
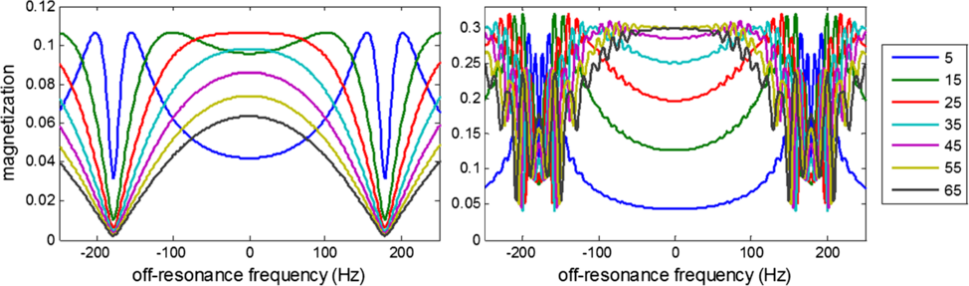
**Off-resonance response for a variety of flip angles for SSFP at steady state (left) and during the approach to steady state (right) for n = 33 RF pulses to k-space center with TR = 2.8 ms and 5 pulse linear run up.** Note that the vertical axes have different scale since the steady state magnetization is reduced.

**Figure 2:**
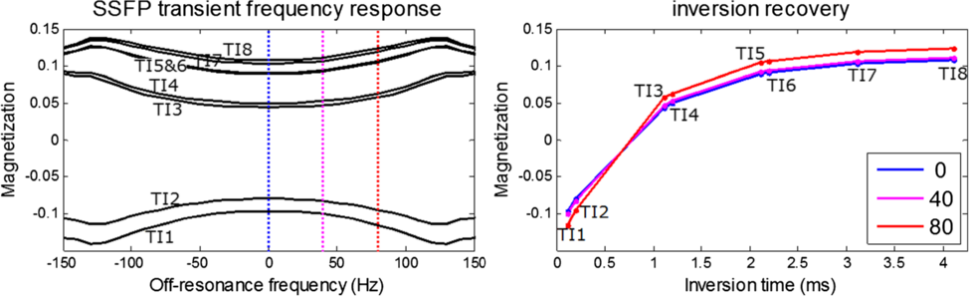
**SSFP off-resonance frequency response during the transient approach to steady state with inversion recovery (left) illustrates that the off-resonance response depends on the initial magnetization thereby influencing the apparent inversion recovery (right) plotted for 0 Hz (blue), 40 Hz (magenta), and 80 Hz (red).** (MOLLI 5(3)3 protocol with FA = 35 deg, TR = 2.8, N = 33 to center, + 5 linear ramp).

Figure 1 shows the response of SSFP readout vs frequency for various flip readout excitation angles shows the familiar dark bands spaced at 1/TR (T1/T2/TR = 1000/45/2.8 ms). The left panel of Figure 1 shows the steady state response and the right panel shows the response for the transient approach to steady state corresponding to single shot imaging with initial full magnetization. The transient response has greater magnetization and different shape off-resonance response curves than the steady state response. The transient approach to steady state for single shot imaging during inversion recovery with different initial magnetization for each inversion time image has an off-resonance response (Figure 2, left) which distorts the apparent T1-recovery (Figure 2 right).

**Figure 3:**
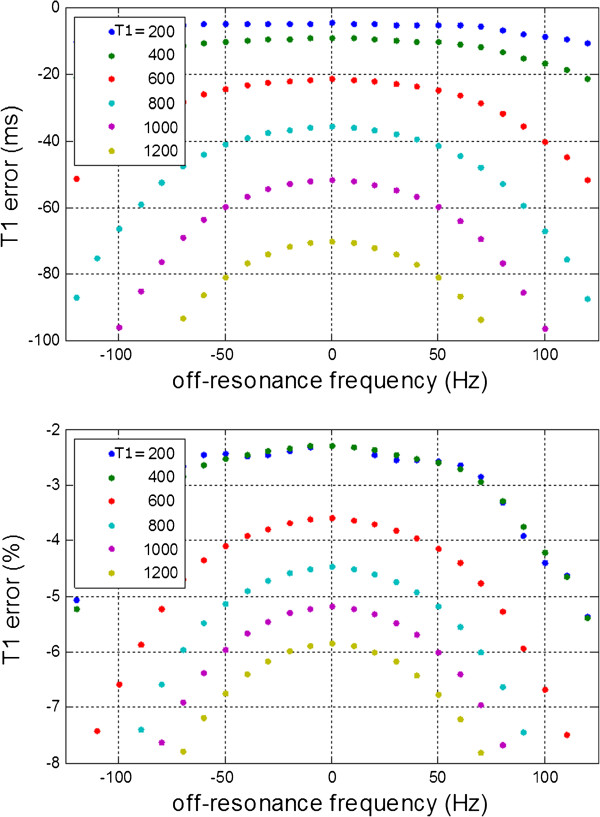
**Off-resonance error in T1 using MOLLI 5(3)3 protocol with 35 degree flip angle for a range of tissue T1 values with T2 = 45 ms, showing error in ms (top) and percent (bottom).** The T1 error and sensitivity to off-resonance frequency increase for higher values of T1.

The off-resonance error in T1 is shown in Figure 3 for a MOLLI 5(3)3 protocol with 35 degree flip angle for a range of tissue T1 values with T2 = 45 ms. Error in ms is displayed on the top panel of Figure 3 and in percent on the bottom panel. The T1 error and sensitivity to off-resonance frequency increase for higher values of T1. At T1=1000 ms, measured T1 varied by 10ms (1%) across ± 50 Hz, and 20 ms (2.0%) across ± 75 Hz. This variation across frequency is in addition to the measurement error for on resonance tissue.

Sensitivity to off-resonance for a nominal T1=1000 ms was simulated for a modified protocol acquired with fewer RF pulses corresponding to smaller matrix size, higher partial Fourier factor, and eliminating in-place auto-calibration. The off-resonance sensitivity at 100 Hz, i.e., difference between on- and off-resonant T1-estimates, was calculated to be 42 ms at 1000 ms (4.2%) using the protocol with Ncenter/Ntotal = 33/75, and reduced to 27 ms (2.7%) using the protocol with Ncenter/Ntotal = 16/48. The off-resonance sensitivity at 50 Hz varied from 0.8% to 0.5%, for the cases 33/75 and 16/48 respectively.

### Phantom measurements

**Figure 4:**
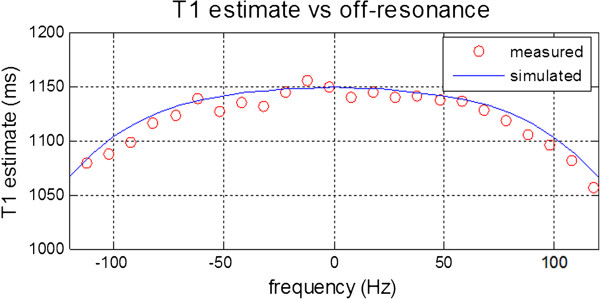
Off-resonance error in T1 comparing phantom measurements (red) with Bloch simulation (blue) for a phantom with T1 = 1197 ms and T2 = 47 ms using MOLLI 5(3s)3 protocol with a 35° flip angle.

Experimental data is in close agreement with the simulation (Figure 4) in the calculation of off-resonance dependence of T1-estimation using MOLLI with SSFP readout.

### In-vivo examples

**Figure 5:**
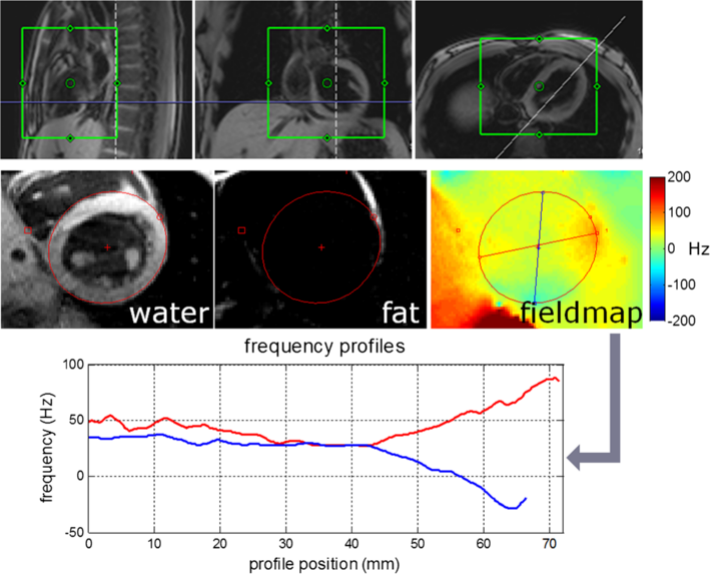
**Local 2**^**nd **^**order shim prescription and field map measurement using multi-echo GRE approach at 3T which jointly estimates water, fat, and field map.** Off-resonance variation is particularly pronounced at the tissue-air interface.

**Figure 6:**
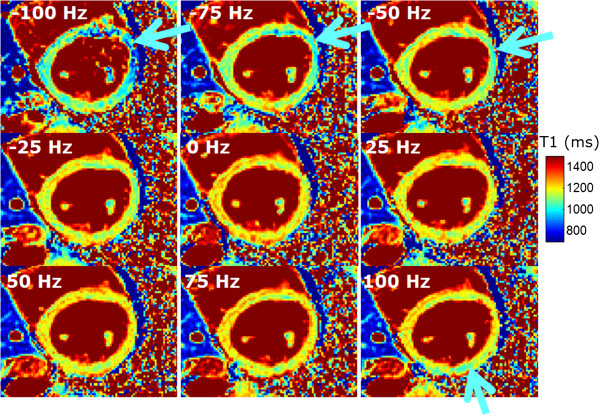
**T1-maps acquired at different center frequencies using MOLLI at 3T.** Despite the use of a 2^nd^ order shim in a local volume around the heart, off-resonant variation across the heart (Figure 5) leads to local variation in the apparent T1 as indicated by arrows.

An example study at 3T demonstrates the sensitivity to off-resonance. In this example, a local 2^nd^ order shim is used (Figure 5) to minimize off-resonance variation. Despite shimming, there is a residual uncompensated off-resonance variation at regions near the tissue-air interface that creates a detectable change in apparent T1 (Figure 6) which is further accentuated with even small errors in center frequency.

**Figure 7:**
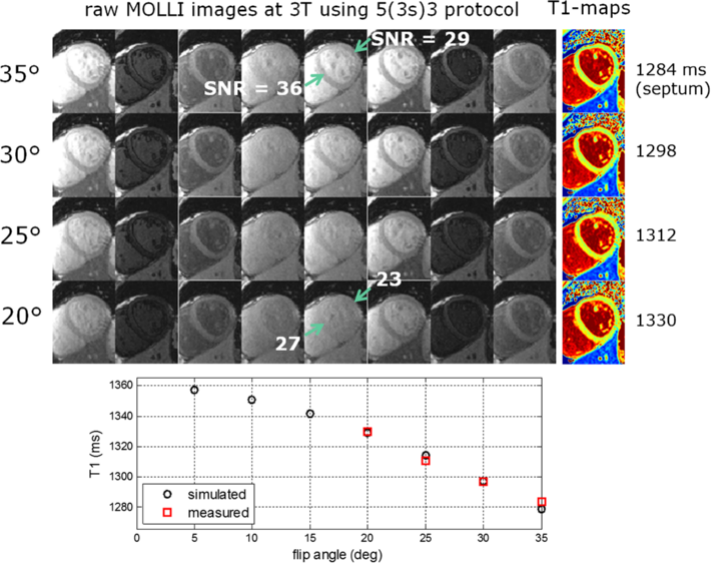
**T1 maps and raw inversion recovery images acquired at 3T using MOLLI 5(3s)3 protocol at various flip angles illustrating tradeoff between SNR and T1-measurement bias.** Simulation of T1 estimates vs measurements using T1 = 1365 ms and T2 = 45 ms.

**Figure 8:**
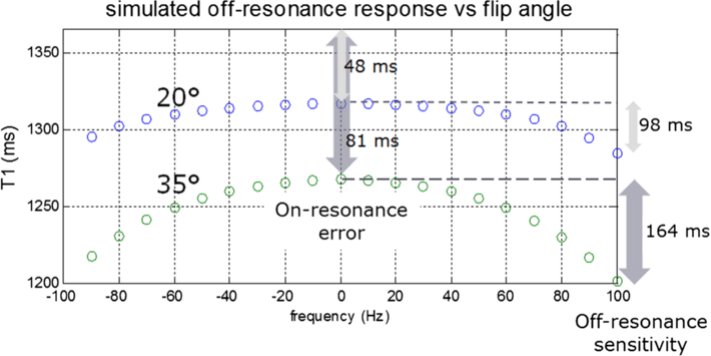
**Using a lower flip angle (FA) trades SNR (precision) for improved accuracy and reduced off-resonance sensitivity.** Simulation compares 20° and 35° readout flip angle for T1 = 1365 ms and T2 = 45 ms (representative values at 3T). T1 bias error due to the effects of readout on the inversion curve is -48 Hz and -81 Hz for FA = 20° and 35°, respectively. Sensitivity to off-resonance over ± 100 Hz is 98 ms and 164 ms for FA = 20° and 35°, respectively.

The example in Figure 7 illustrates the trade-off between SNR and T1-measurement bias by acquiring data at different flip angles. Simulation of T1 estimates vs. measurements using T1 = 1365 ms and T2 = 45 ms. Using a lower flip angle (FA) trades SNR (precision) for improved accuracy and reduced off-resonance sensitivity. Reducing the flip angle from 35° to 20° causes a reduction in SNR from 36 to 27 in the septum and 29 to 23 in the lateral wall. The T1 estimate in the septum is 1284 ms at 35° and 1330 at 20° which agrees well with simulation (Figure 7) using T1/T2 = 1365/45 ms. The T1 measurement error off-resonance is calculated by simulation (Figure 8) for this example. T1 bias error due to the effect of the readout on the inversion recovery curve is -48 ms and -81 ms for FA = 20° and 35°, respectively. Sensitivity to off-resonance over ± 100 Hz is 98 ms and 164 ms for FA = 20° and 35°, respectively.

## Discussion

### T1 underestimation

It is generally assumed that shimming and center frequency adjustment are not significant problems at 1.5T. Maximum off-resonance averaged 61 ± 15.5 Hz across the LV (n = 18) leading to an apparent variation in T1 of 0.5% for T1 = 400 ms, and > 1% for T1 = 1000 ms (calculated using a Bloch simulation for the specific MOLLI protocol described). This represents the expected mean error in T1 (n = 18), however, off-resonance > 80 Hz was observed in 4 of 18 subjects, which resulted in more significant T1 errors (> 3%) which could be confused with regional variation in T1 due to pathology. In a large study of normal subjects [20], the T1 measurements were reported to have standard deviation < 2% at 1.5T. However, that study measured the mean per subject across the heart which tends to downplay regional variation due to local susceptibility. At 3.0T the off-resonance is typically greater, with mean off-resonance found to be ~125 Hz across the LV, after local shimming. The variation in T1 at 3T from on-resonance with this protocol was calculated to be approximately 3% at 125 Hz for T1 = 400 ms, and > 5% at 125 Hz for T1 = 1000 ms. Off-resonance >150 Hz was observed (7 of 18 subjects) leading to significant regional variation.

In this study, the readout size was 256 sampled in the frequency direction in order to improve the spatial resolution to help mitigate partial volume effects at tissue interfaces, e.g, myocardium and blood. This readout length results in a TR of 2.8 ms. A shorter TR (and readout) would somewhat decrease the sensitivity to off-resonance. The off-resonance sensitivity is also dependent on the number of RF pulses to the center of k-space and total number of readouts, and can be reduced by decreasing the matrix size and greater partial Fourier. Modifying the protocol in this way may result in lower spatial resolution and degraded point spread function leading to T1-errors caused by greater partial volume contamination of the myocardium by the adjacent blood pool.

The use of separate reference line auto-calibration of parallel imaging eliminates 12 phase encodes per shot (6 to center of k-space) using the current widely used in-place calibration approach. For this reason, we have recently adopted a separate reference line approach which acquires the auto-calibration data at the end of the imaging acquisition such that the reference lines do not influence the initial magnetization. This has the additional benefit of reducing the overall shot time by 34 ms which is particularly important at higher heart rates to reduce cardiac motion blur.

Reduction in excitation flip angle can reduce the off-resonance sensitivity at the cost of decreased SNR. Reduction in flip angle also reduces the absolute bias error (i.e., on-resonance error). Using a FA = 35° the SNR is in the range 20-40 across the LV and the precision of T1 estimates is between 45 and 22 ms (SD measured on a per pixel basis), respectively, using the 5(3)3 protocol [21]. By reducing the flip angle from 35° to 20° the SNR is reduced by approximately 40% to the range of 15-30 across the LV. With this SNR reduction, the precision of T1 estimates is 60 to 30 ms, respectively. The precision of the T1 estimated in a myocardial sector is improved several fold by averaging the voxels within the sector, however, per pixel precision is still important for detecting subtle regional variation. This trade-off might be worthwhile particularly at 3T where there is greater SNR to begin with and the magnitude of the off-resonance problem is expected to be greater. At some point, a FLASH based readout also becomes competitive but this requires further optimization, which is beyond the scope of this study.

### Off-resonance variation

Off-resonance maps may be useful for improved confidence in measured T1-values, although it would be difficult to correct the T1 values. Values for typical off-resonance variation were established in a relatively small patient population where the variation is primarily caused by tissue air interfaces. In subjects with sternal wires or devices the off-resonance variation may increase. In severe cases with rapid spatial variation of off-resonance, an additional issue may arise in cases where the subject has difficulty breath-holding. In this case, the possibility arises that the off-resonance variation over time will also affect the estimate of T1, but this case has not been studied.

Lastly, since it has been shown that the sensitivity to off-resonance is a function of flip angle, there is a complex interaction between the variation in off-resonance due to B0-inhomogeneity and the variation in actual flip angle due to B1 transmit inhomogeneity. The B0 and B1+ field inhomogeneities are fairly independent, however sensitivity of T1 estimate to variation in flip angle has not been studied.

### Off-resonance sensitivity of ECV

Errors in T1 due to off-resonance may also influence the calculation of ECV maps [10] derived from T1-maps. A preliminary analysis has shown that the systematic bias errors in ECV due to off-resonance is quite low, on the order of 1% or less (in units of ECV percentage). Although the blood T1 is much longer, errors in ECV are dominated by the pre-contrast T1. Interestingly, the T1 measurement blood is not sensitive to off-resonance as a result of flow. The source of error in the stationary myocardial tissue T1 estimate using the MOLLI method is due to the approximation of the so called Look-Locker correction (B/A-1) which arises due to modification of the apparent inversion recovery curve leading to a T1* that is shorter than T1. In the case of flowing blood where there are new spins at each beat, the inversion recovery is not influenced by the previous readout and the T1* from the 3-parameter exponential fit provides and an accurate estimate of T1.

## Conclusions

The use of single shot SSFP imaging for T1-mapping in the heart is accompanied by an off-resonance sensitivity of the T1 estimates. Significant T1 measurement errors may arise well within the SSFP “passband” in regions that do not experience dark band artifacts. The off-resonant behavior is due to the transient approach to steady state, which depends on the initial magnetization determined by the inversion recovery. The apparent T1 depends on off-resonance leading to a T1-underestimation. Off resonance errors worsen with higher excitation flip angles. Regional variations due to the inability to completely shim the B0-field variation around the heart appear as regional variation in T1, which is artifactual.

## Abbreviations

CMR: Cardiovascular Magnetic Resonance; MOLLI: modified Look-Locker inversion recovery; TI: Inversion time; ROI: Region-of-interest.

## Competing interests

Dr. Arai is a principal investigator on a US government Cooperative Research and Development Agreement (CRADA) with Siemens Medical Solutions (HL-CR-05-004).

## Authors’ contributions

PK conceived of the study, wrote the simulation, performed measurements and analysis, and drafted the manuscript. DAH contributed to the Bloch simulations. AEA had overall responsibility for human studies, MSH contributed to the interpretation of results. All authors participated in revising the manuscript and read and approved the final manuscript.

## References

[B1] Bieri O Scheffler K Fundamentals of balanced steady state free precession MRI J Magn Reson Imaging 2013 [Epub ahead of print] 10.1002/jmri.24163 23633246

[B2] Wieben O Francois C Reeder SB Cardiac MRI of ischemic heart disease at 3T: potential and challenges Eur J Radiology 1008 65 1 15 28 10.1016/j.ejrad.2007.10.022 18077119

[B3] Schar M Kozerke S Fischer SE Boesiger P Cardiac SSFP imaging at 3 Tesla Magn Reson Med 2004 51 799 806 10.1002/mrm.20024 15065254

[B4] Messroghli DR Radjenovic A Kozerke S Higgins DM Sivananthan MU Ridgway JP Modified look-locker inversion recovery (MOLLI) for highresolution T1 mapping of the heart Magn Reson Med 2004 52 141 146 10.1002/mrm.20110 15236377

[B5] Messroghli DR Greiser A Frohlich M Dietz R Schulz-Menger J Optimization and validation of a fully-integrated pulse sequence for modified look-locker inversion-recovery (MOLLI) T1 mapping of the heart J Magn Reson Imaging 2007 26 1081 6 10.1002/jmri.21119 17896383

[B6] Flett AS Hayward MP Ashworth MT Equilibrium contrast cardiovascular magnetic resonance for the measurement of diffuse myocardial fibrosis: preliminary validation in humans Circulation 2010 122 2 138 144 10.1161/CIRCULATIONAHA.109.930636 20585010

[B7] Sado DM Flett AS Moon JC Novel imaging techniques for diffuse myocardial fibrosis Future Cardiol 2011 7 5 643 50 10.2217/fca.11.45 21929344

[B8] Schelbert E Testa SM Meier CG Myocardial extracellular volume fraction measurement by gadolinium cardiovascular magnetic resonance in humans: slow infusion versus bolus J Cardiovasc Magn Reson 2011 13 16 10.1186/1532-429X-13-16 21375743 PMC3059279

[B9] Ugander M Oki AJ Hsu L-Y Extracellular volume imaging by MRI provides insight into overt and subclinical myocardial pathology Eur Heart J 2012 33 10 1268 78 10.1093/eurheartj/ehr481 22279111 PMC3350985

[B10] Kellman P Wilson JR Xue H Ugander M Arai AE Extracellular volume fraction mapping in the myocardium, Part 1: evaluation of an automated method J Cardiovasc Magn Reson 2012 14 63 10.1186/1532-429X-14-63 22963517 PMC3441905

[B11] Kellman P Wilson JR Xue H Bandettini WP Shanbhag SM Druey KM Ugander M Arai AE Extracellular volume fraction mapping in the myocardium, Part 2: initial Clinical Experience J Cardiovasc Magn Reson 2012 14 64 10.1186/1532-429X-14-64 22967246 PMC3442966

[B12] Karamitsos TD Piechnik SK Banypersad SM Noncontrast T1 mapping for the diagnosis of cardiac amyloidosis JACC Cardiovasc Imaging 2013 13 00138 1 pii: S1936-878X, [Epub ahead of print] 10.1016/j.jcmg.2012.11.013 23498672

[B13] Bull S White SK Piechnik SK Human non-contrast T1 values and correlation with histology in diffuse fibrosis Heart 2013 [Epub ahead of print] 10.1136/heartjnl-2012-303052 PMC3686317 23349348

[B14] Sado DM White SK Piechnik SK The identification and assessment of Anderson fabry disease by cardiovascular magnetic resonance non-contrast myocardial T1 mapping Circ Cardiovasc Imaging 2013 [Epub ahead of print] 10.1161/CIRCIMAGING.112.000070 23564562

[B15] Deichmann R Haase A Quantification of Tl Values by SNAPSHOT-FLASH NMR Imaging J Magn Reson 1992 612 608 612

[B16] Gai ND Stehning C Nacif M Bluemke DA Modified Look-Locker T(1) evaluation using Bloch simulations: Human and phantom validation Magn Res Med 2012 [Epub ahead of print] 10.1002/mrm.24251 PMC3826815 22457268

[B17] Kellman P Herzka DA Hansen MS Adiabatic inversion pulses for myocardial T1-mapping Magn Res Med 2013 [Epub ahead of print] 10.1002/mrm.24793 PMC3775900 23722695

[B18] Deshpande VS Chung YC Zhang Q Shea SM Li D Reduction of transient signal oscillations in True-FISP using a linear flip angle series magnetization preparation Magn Reson Med 2003 49 151 157 10.1002/mrm.10337 12509831

[B19] Hernando D Kellman P Haldar JP Liang Z-P Robust water/fat separation in the presence of large field inhomogeneities using a graph cut algorithm Magn Reson Med 2010 63 1 79 90 19859956 10.1002/mrm.22177 PMC3414226

[B20] Piechnik SK Ferreira VM Lewandowski AJ Ntusi NA Banerjee R Holloway C Hofman MB Sado DM Maestrini V White SK Lazdam M Karamitsos T Moon JC Neubauer S Leeson P Robson MD Normal variation of magnetic resonance T1 relaxation times in the human population at 1.5 T using ShMOLLI J Cardiovasc Magn Reson 2013 15 13 10.1186/1532-429X-15-13 23331520 PMC3610210

[B21] Kellman P Arai AE Xue H T1 and extracellular volume mapping in the heart: estimation of error maps and the influence of noise on precision J Cardiovasc Magn Reson 2013 15 1 56 10.1186/1532-429X-15-56 23800276 PMC3702513

